# Collagenase Administration into Periodontal Ligament Reduces the Forces Required for Tooth Extraction in an Ex situ Porcine Jaw Model

**DOI:** 10.3390/jfb13020076

**Published:** 2022-06-08

**Authors:** Ran Tohar, Hen Alali, Tamar Ansbacher, Tamar Brosh, Inbal Sher, Yossi Gafni, Evgeny Weinberg, Maayan Gal

**Affiliations:** 1 Department of Oral Biology, Goldschleger School of Dental Medicine, Faculty of Medicine, Tel Aviv University, Tel Aviv 6997801, Israel; tohar.ran@gmail.com (R.T.); henalali@mail.tau.ac.il (H.A.); tamar.ansbacher@mail.huji.ac.il (T.A.); tbrosh@tauex.tau.ac.il (T.B.); inbalasher87@gmail.com (I.S.); 2 Hadassah Academic College, Jerusalem 91010, Israel; 3 Department of Orthodontics, Goldschleger School of Dental Medicine, Faculty of Medicine, Tel Aviv University, Tel Aviv 6997801, Israel; drgafni@gmail.com; 4 Department of Periodontology and Oral Implantology, Goldschleger School of Dental Medicine, Faculty of Medicine, Tel Aviv University, Tel Aviv 6997801, Israel

**Keywords:** exodontia, collagen, collagenase, minimally invasive medicine, periodontal ligament

## Abstract

Minimally invasive exodontia is among the long-sought-for development aims of safe dental medicine. In this paper, we aim, for the first time, to examine whether the enzymatic disruption of the periodontal ligament fibers reduces the force required for tooth extraction. To this end, recombinantly expressed clostridial collagenase G variant purified from Escherichia coli was injected into the periodontal ligament of mesial and distal roots of the first and second split porcine mandibular premolars. The vehicle solution was injected into the corresponding roots on the contralateral side. Following sixteen hours, the treated mandibles were mounted on a loading machine to measure the extraction force. In addition, the effect of the enzyme on the viability of different cell types was evaluated. An average reduction of 20% in the applied force (albeit with a large variability of 50 to 370 newton) was observed for the enzymatically treated roots, reaching up to 50% reduction in some cases. Importantly, the enzyme showed only a minor and transient effect on cellular viability, without any signs of toxicity. Using an innovative model enabling the analytical measurement of extraction forces, we show, for the first time, that the enzymatic disruption of periodontal ligament fibers substantially reduces the force required for tooth extraction. This novel technique brings us closer to atraumatic exodontia, potentially reducing intra- and post-operative complications and facilitating subsequent implant placement. The development of novel enzymes with enhanced activity may further simplify the tooth extraction process and present additional clinical relevance for the broad range of implications in the oral cavity.

## 1. Introduction

Tooth extraction (i.e., exodontia) is a fundamental procedure performed in dental surgery [1,2]. Ultimately, exodontia should enable controlled and safe tooth removal, followed by complete healing without post-operative complications. The basic principles of tooth extraction include the disruption of periodontal ligament (PDL) fibers from the bundle bone of the socket and primary tooth luxation, usually achieved by employing a dental elevator, followed by further socket expansion and delivery of the tooth with dental forceps [2,3]. In some cases, to successfully remove the tooth, the reflection of a mucoperiosteal flap and removal of the alveolar bone are unavoidable to visualize and gain access to the roots of teeth. Occasionally, root separation is required as well. Therefore, tooth extraction is an invasive procedure, often characterized by difficult to avoid collateral damage to surrounding soft and hard tissues. Among the various intra- and post-operative complications are root fracture, bleeding and hemorrhage, displaced teeth, bone fracture, soft tissue injury, damage to an adjacent tooth, infection, alveolar osteitis, and paresthesia [4,5,6,7,8,9]. Moreover, tooth extraction is associated with osteonecrosis of the jaw in patients with a history of antiresorptive (e.g., bisphosphonates) or antiangiogenic agents [10,11]. Furthermore, rehabilitation with dental implants may become more complex whenever hard and soft tissues are not preserved during the tooth extraction [12,13]. Atraumatic or minimally invasive exodontia can reduce intra- and post-operative complications and facilitate the subsequent implant placement and restoration by reducing the damage to soft and hard tissues surrounding the tooth being extracted.

Together with additional breakthroughs in many other branches of dentistry, technological advancements in exodontia showed constant improvements in extraction techniques and instruments [12,14,15,16,17]. However, the current techniques of minimally invasive tooth extraction either implement mechanical disruption of PDL fibers, such as periotomes and piezosurgery [12,18,19,20], or limit the extraction forces to the vertical dimension (e.g., the Benex^®^ extraction system), thus minimizing socket expansion [15,21]. An additional tool designed for atraumatic tooth extraction is a physics forceps, which decreases the risk of fractures in the crown, root, and buccal bone plate compared to using conventional forceps [14]. However, no generic solution exists that actually reduces the physical force required for tooth extraction.

Collagen is considered the main structural component of PDL, anchoring the tooth to the bundle bone [22]. Hence, a promising approach for minimally invasive tooth extraction is the degradation of the collagen bundles. The family of collagenases comprise the natural machinery for cleaving collagen. These enzymes are an essential part of the matrix metalloproteinase family of proteins, and their main function is to regulate the matrix through the degradation of collagen [23]. Different types of collagenases are produced by both mammalian cells and bacteria. The latter are known as efficient enzymes capable of degrading triple-helix collagen and digesting it down to short peptide fragments [24]. Indeed, given its efficient activity, the collagenase G of Clostridium histolyticum is approved for the treatment of Dupuytren’s and Peyronie’s diseases [25,26], as well as for the treatment of burns and wounds [27].

Based on the imperative role of collagen in the PDL and the efficient activity of collagenase enzymes, the aim of the present study is to investigate for the first time whether the enzymatic disruption of the PDL fibers can actually reduce the force required for tooth extraction. Indeed, collagenase was previously shown to alter the area of collagen fibers in the PDL [22,28]. In this paper, we present an innovative approach to the analytical monitoring of extraction force, which shows clear evidence for a substantial reduction in the force required for tooth extraction following the enzymatic-based disruption of PDL fibers with collagenase in an ex situ model of porcine jaw.

## 2. Materials and Methods

### 2.1. Expression and Purification of Collagenase G

The expression of recombinant collagenase G (ColG) was performed as described elsewhere [29]. Briefly, the plasmid-containing ColG gene derived from *Clostridium histolyticum*, comprising amino acids 119–1118 with a tobacco etch virus (TEV) cleavable N-terminus Hisx6 tag, was transformed into *E. coli* BL21. Cells were grown to OD (600 nm) = 0.8, and protein expression was induced by supplementing 1 mM Isopropyl β-d-1-thiogalactopyranoside (IPTG) for 16 h at 25 °C. Following the lysis of bacterial cells, the supernatant was passed onto a nickel column, and following extensive wash, the protein was eluted with 300 mM imidazole. Following buffer exchange to phosphate-buffered saline (PBS), ColG was kept frozen with the addition of 50% glycerol.

### 2.2. Experimental Set-Up and Jaw Preparation

**Figure 1:**
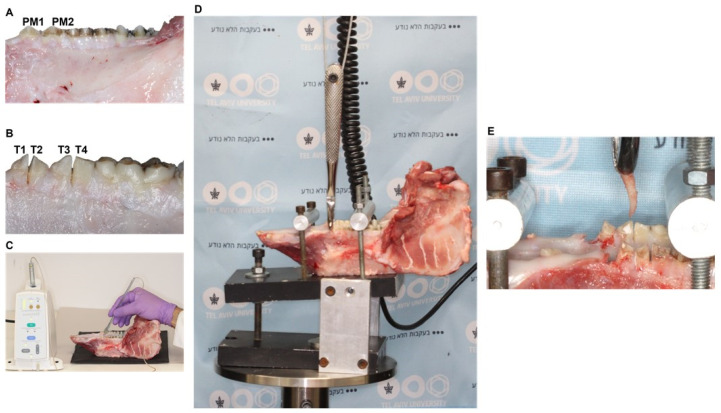
**Preparation of the porcine jaw for tooth extraction.** (**A**) Left side of the lower jaw after the removal of soft tissues. Premolar teeth are marked as PM1 and PM2. (**B**) After root splitting, the roots are marked as T1 (mesial root of PM1), T2 (distal root of PM1), T3 (mesial root of PM2), and T4 (distal root of PM2). (**C**) Injection of collagenase using a computer-assisted injection system; (**D**) The mandible set in the self-designed anchoring device and mounted on a loading machine. (**E**) The root after extraction.

The whole mandibles of six-month-old (90–100 kg) domestic swine were obtained from a local abattoir (Marsel Brothers Company, Haifa, Israel) close to slaughter. Only healthy samples with unimpaired teeth, gingival tissues, and alveolar mucosa were selected. A total of twelve jaws were chosen for the experiments on the first and second premolar teeth (PM1 and PM2, respectively), each containing two divergent roots, mesial and distal. The jaws were carefully debrided from the soft tissues, including gingiva adjacent to the teeth, thus exposing the tooth roots up to the alveolar crest (Figure 1A). Before the injection of either ColG or the vehicle solution, the mesial and distal roots of PM1 and PM2 were split using a dental handpiece and high-speed diamond bur (Strauss & Co, Ra’anana, Israel). These are marked as T1 (mesial root of PM1), T2 (distal root of PM1), T3 (mesial root of PM2), and T4 (distal root of PM2) in Figure 1B. Thereafter, mandibles were randomly divided by a split-mouth design into two sides, ColG and PBS (a vehicle of ColG that served as a negative control).

### 2.3. Injection of Collagenase into the PDL

Either collagenase or PBS were injected into the PDL using a computer-assisted injection system (Wand Single Tooth Anesthesia system, Milestone Scientific, Roseland, NJ, USA) (Figure 1C), which enables a controllable and precise injection through a clinically accepted approach. Briefly, standard cartridges containing the local anesthetic solution for dental injection were accurately emptied of their content and filled with either ColG at a concentration of 4 µg/µL or PBS, followed by application to each of the examined roots in a split-mouth design. Injection was performed using a 30 G 2.54 cm length needle that was inserted into the PDL space and advanced apically until stopped by the resistance of the alveolar bone proper. The injection was repeated at four sites around each root, on the buccal, lingual, mesial, and distal aspects. A total of 0.3 mL of ColG or PBS was injected overall per root. After an incubation period of sixteen hours at room temperature, which was intended to enable maximum enzymatic activity, the extraction forces were measured as described below.

### 2.4. Mounting of Porcine Mandibles to the Loading Machine

A device anchoring to the lower jaw of the loading machine (Instron Series 4500; Instron Corp., Canton, MA, USA) was specifically self-designed, with two parallel height-adjustable cylinders for securing the anterior and posterior mandible relative to the tooth root to be extracted (Figure 1D). The device was designed to enable the fixation of the porcine mandible under varying inclinations, so that the longitudinal axes of the tooth root could be adjusted perpendicularly to the ground surface, thus limiting the extraction forces to the vertical dimension. Straight upper incisor and canine extraction forceps (Hu Friedy, Chicago, IL, USA), with drilled holes in the distal edges of the handles and a pin connecting them, were attached to the upper jaw of the loading machine via a pull rope and locked firmly over the clinical crown of the root to be extracted.

### 2.5. Measurement of Force Required for Root Extraction

The extraction force was applied to each of the examined roots by the loading machine using a load cell of 10 kilonewtons and a crosshead speed of 10 mm/min, until the root was completely removed from the alveolar socket (Figure 1E). The assessment of the tensile force and displacement during the root extraction process was achieved via the designated software (Instron Series IX; Instron Corp., Canton, MA, USA) at a rate of 10 hertz. One mesial and one distal root of PM1 (T1 and T2) broke prematurely and were excluded from the data, as were their contralateral counterparts. Therefore, data regarding the extraction force were obtained for the following number of the corresponding contralateral root pairs: eleven T1, eleven T2, twelve T3, and twelve T4.

### 2.6. Cellular Viability Assay

Chinese Hamster Ovary (CHO) cells were obtained from the lab of Rina Rosin-Arbesfeld [30]. Cells were cultured in a growth medium containing Dulbecco’s Modified Eagle Medium (DMEM) supplemented with 10% fetal calf serum (FCS), 2 mM glutamine, 100 U/mL penicillin, 100 mg/mL streptomycin, 12.5 U/mL nystatin, 0.11 mg/mL sodium pyruvate, and non-essential amino acids (Biological Industries, Beit HaEmek, Israel) at 37 °C in a humidified atmosphere of 5% CO_2_ and 95% air.

Experiments with primary human gingival fibroblasts (hGFs) were approved by the Tel Aviv University institutional review board (IEC No. 0001006-1). Informed consent was obtained from all patients. Fragments of healthy masticatory oral mucosa collected during periodontal surgeries were harvested from free Caucasian donors. The inclusion criteria were non-smoking status and absence of any systemic disease. Exclusion criteria consisted of pregnant and lactating women and a history of periodontal disease. Cells were separated into connective tissue and epithelium using dispase II (Sigma-Aldrich, Rehovot, Israel) at 2 mg/mL for two hours at 37 °C. Subsequently, the connective tissue was cut into smaller pieces, which were then placed in the same growth medium as CHO cells and under the same conditions, to allow cell outgrowth. The medium was replaced every three days until confluence was reached. Cells of the second or third passage were used for experiments.

For the viability assay, either CHO cells or hGFs were seeded in a 96-well plate, 40,000 cells/well in 200 μL of growth medium with ColG at different concentrations ranging from 5 µg/mL up to 5 mg/mL. The cells were incubated overnight at 37 °C in a humidified atmosphere of 5% CO_2_ and 95% air. To assess cellular viability, 100 μL media was removed and replaced with 100 μL of cell viability reagents (CellTiter, Promega, Madison, WI, USA), evaluating the nucleotide adenosine triphosphate (ATP) levels to monitor viable and metabolically active cells. The plate was placed in a BioTek SynergyH (Agilent, Santa Clara, CA, USA) plate reader and luminescence values were read, according to manufacturer’s instructions. Data were normalized based on the read values of the PBS-treated cells.

### 2.7. Cellular Toxicity

The fluorescence live–dead staining assay (Sigma-Aldrich, Rehovot, Israel) containing fluorescein diacetate (6.6 μg/mL) and propidium iodide (5 μg/mL) was used to visualize the viable and dead cells. Cell images were taken using a ZEISS LSM 900, Laser Scanning Microscope. Fluorescence readouts were obtained with λex/λem of 490/515 nm and 535/617 nm for green and red, respectively.

### 2.8. Data Analysis

The data were analyzed and the figures were plotted with GraphPad prism 7 (RRID:SCR_002798). *p*-values were calculated using a paired *t*-test with Gaussian distribution.

## 3. Results

### 3.1. Experimental Setup

As a first step, we embarked on setting a standardized model for the direct analytical measurement of the force required for tooth extraction. Our approach was based on an ex situ porcine jaw, which is recognized as a validated model for a range of dental applications [31]. Figure 1 summarizes the entire process, from jaw preparation to the root extraction with a loading machine. Our preliminary analysis revealed that, owing to anatomical properties, such as considerable root divergence and fragility of the examined teeth, splitting the premolars comprises an essential step to reduce the extraction force to a strength comparable to that of humans [32]. Otherwise, the magnitude of the tooth extraction force in the porcine jaw resulted in premature root or crown fracture. Furthermore, the splitting of PM1 and PM2 allowed us to set the self-designed anchoring device under the inclination that limited the extraction force to the vertical dimension, along the longitudinal axes of each extracted root. All these measures enabled us to pull out separately the intact mesial and distal roots of PM1 and PM2 from their sockets, while recording the extraction force.

### 3.2. Qualitative and Quantitative Analysis of Extraction Forces

**Figure 2:**
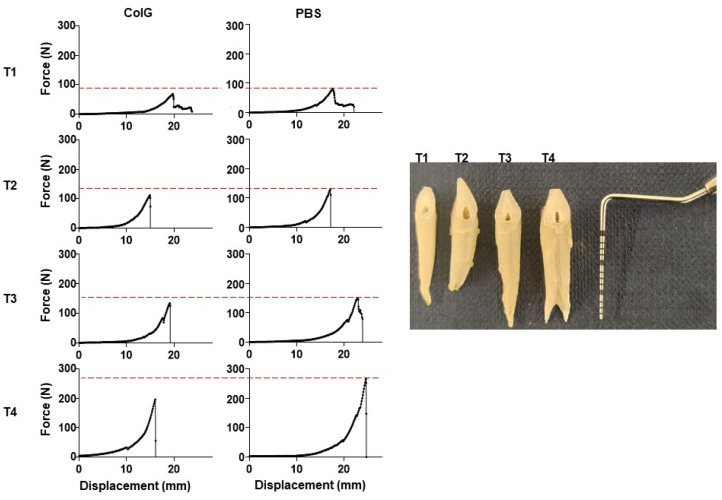
**Real-time recording of force and displacement during tooth extraction**. For each root following injection of ColG or PBS, the force in newton (N) vs. displacement (mm) was recorded during extraction. Four exemplified extraction curves are shown for roots T1, T2, T3, and T4. The red-dashed curve marks the highest recorded force in the PBS-treated roots. The right panel shows a picture of four extracted roots. The scale of the probe is 1 mm per bar.

In the next step, we turned to measure the extraction forces of either T1, T2, T3, or T4 (Figure 1B) by the real-time recording of the displacement of each examined root vs. the applied force. Figure 2 shows representative curves of force versus distance for each of the four pairs of roots that were extracted after the application of ColG or PBS. A different distance at which the pulling cable reached the point of complete stretch and started to pull the tooth is observed in each of the curves; this yielded an apparently varied distance for the different roots. We could distinguish between several extraction patterns (Figure 2). Evidently, the representative curves reveal that T1 has the lowest maximum extraction force. In addition, T1 showed a unique pattern, whereby following the maximal peak after which the root was extracted, the force did not immediately drop to zero, but rather fell to a secondary baseline. This suggests that, following root extraction from the PDL, soft tissues still connect the root to the alveolar ridge, probably due to non-complete removal during the jaw preparation. Roots T2 and T3 demonstrated an overall similar extraction pattern, with a comparable maximal pulling force. T4’s extraction required the application of the greatest maximal force. In addition to the pattern shown in Figure 2, an additional pattern was observed in several T4 roots. This included an additional lower peak following the main peak that occurred due to proximity-related friction of the T4 crown to the first molar tooth. It is enlightening to view the results in the context of the structural properties of the roots. The right panel in Figure 2 shows the anatomical diversities between representative roots. Root T1 is the narrowest, but is longer than T2. The low surface area resulted in a relatively low extraction force. Indeed, although root T2 is the shortest, it is wider than T1 and of a similar width to T3, but has two fused canals, thus showing comparable force to the longer T3. Root T4 is the widest, with two distinguished separated canals. Accordingly, T4 required the application of the greatest extraction force.

**Figure 3:**
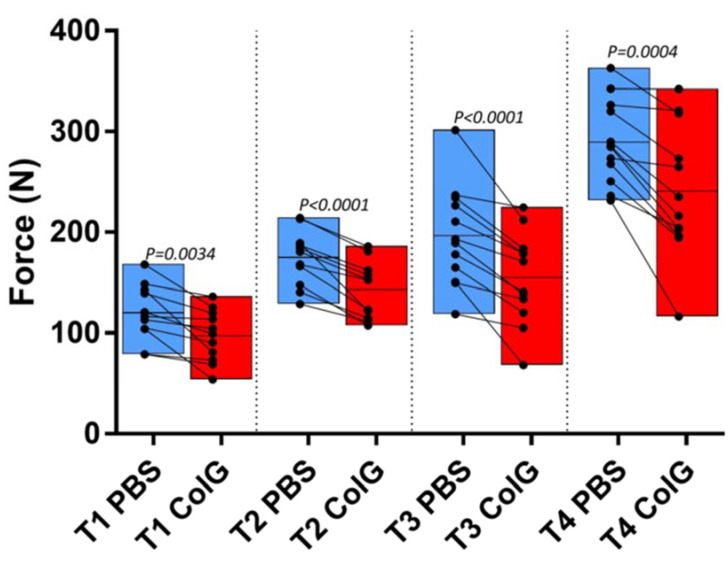
**Mean and dispersion of extraction forces of ColG- and PBS-treated roots.** The blue and red boxes show treatment with PBS and ColG, respectively. Specific measurements are marked by filled circles, and the mean is marked by the horizontal line. Lines connect roots within the same jaw that were treated with ColG and PBS. Statistically significant values are indicated above each paired data.

To evaluate the effect of the injected ColG on the extraction force of the different roots, we calculated the mean extraction force for each root type. Figure 3 shows the mean force (marked by a horizontal black line), as well as the values and dispersion of the root-specific maximal force applied to extract T1–4 in all jaws following treatment with PBS (blue) or ColG (red). A clear reduction in the mean force following treatment with ColG relative to PBS is observed for each root type. Despite the clear reduction in force, the dispersion of maximum force of the same root type in the different jaws was relatively high. This is due to the considerably large degree of variability in the physiological and morphological characteristics of PDL, thus corroborating observations in human jaws [15,32].

**Figure 4:**
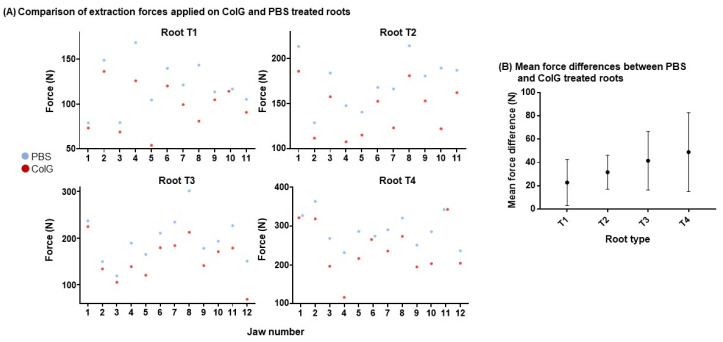
**Comparison of extraction forces applied to ColG- and PBS-treated roots**. (**A**) Each panel represents a different type of root, T1–T4. Vertical dots show extraction forces for roots in the same jaw, which were treated with ColG (red) or PBS (blue). (**B**) For each root, the extraction force of the ColG-treated root was reduced in comparison to that of the PBS-treated root in the same jaw. The calculated mean of the differences for T1–4 is shown.

We then inspected the force of the ColG- vs. PBS-treated contralateral roots in the same jaw. Figure 4A shows the maximal extraction force for T1 (top left), T2 (top right), T3 (bottom left), and T4 (bottom right) across the different jaws. An overall reduction in the applied force was observed in almost all the roots. In some roots, a 50% reduction in the required force was recorded (e.g., T4 in jaw #4 and T1 in jaw #8). However, in a minority of incidences, only negligible differences were detected (e.g., T1 in jaw#10, T4 in jaw#1, and T4 in jaw#11). Of the 44 extracted roots, 20 showed >20% reduction in the applied force following treatment with ColG. Only five tooth roots showed <5% difference (Figure 4B). Roots with only slight differences in force between the ColG and PBS treatments were typically part of the same jaw (e.g., jaw #1). Figure 4B shows the mean difference in the applied extraction force between paired roots, treated with ColG versus PBS, for all jaws. Although the standard error was relatively high, a clear and clinically significant reduction in force was observed following the application of the enzyme ColG.

### 3.3. Evaluation of Cellular Viability

**Figure 5:**
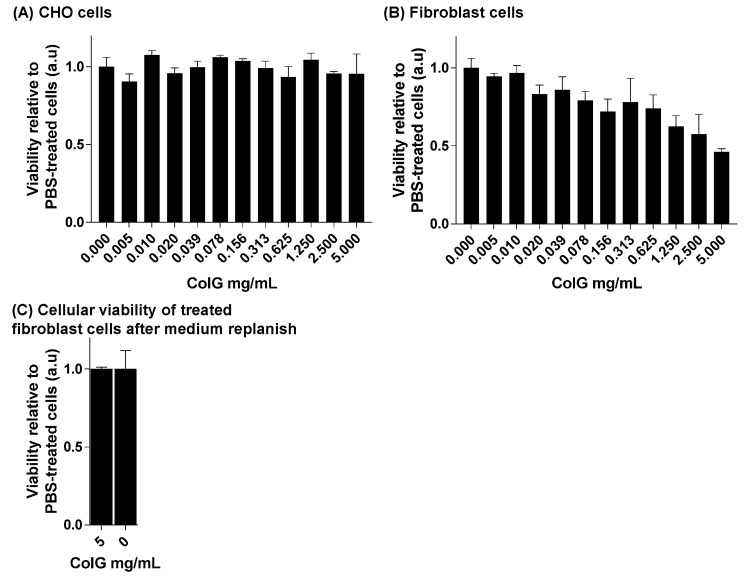
**Cellular viability of CHO cells and hGFs treated with ColG**. (**A**) CHO and (**B**) primary human gingival fibroblast viability. Cells were treated with variable concentrations of ColG, and cellular viability was evaluated. Results are shown relative to PBS-treated cells. (**C**) Cells that were treated with PBS or 5 mg/mL ColG were re-plated with a fresh medium, and cellular viability was evaluated again. Results are relative to cells originating from PBS-treated cells.

In addition to the effect the enzyme has on the force required for the tooth extraction, it is imperative to test parameters related to its safety. Thus, we evaluated the effect of ColG on the viability of non-collagen-dependent and collagen-dependent cells. Figure 5A shows the viability of CHO cells treated with variable ColG concentrations relative to PBS-treated cells. The number of viable cells stays at the same level and does not depend on ColG concentrations, thus demonstrating the safety of the ColG on non-collagen-dependent cells, such as CHO. Of particular interest is the effect of the enzyme on the principal cells inhabiting the gingiva and the mucosal tissue located in close proximity to the site of injection. For this reason, we tested the viability of hGFs following treatment with ColG, with identical concentrations as applied to the CHO cells (Figure 5). As expected from other collagen-dependent cells [33], the degradation of collagen due to ColG activity hampered the hGF vitality in a dose-dependent manner. However, we assumed that the effect of collagenase on hGFs is merely transient and that the growth rate would return to normal once ColG is depleted from the medium. To validate this, 40,000 cells that had been treated with PBS or with the highest ColG concentration (5 mg/mL) were re-plated with a fresh medium that did not contain ColG. Following 24 h, the viability of hGFs was measured again. Figure 5C shows that the viability of ColG- and PBS-pre-treated cells is similar.

**Figure 6:**
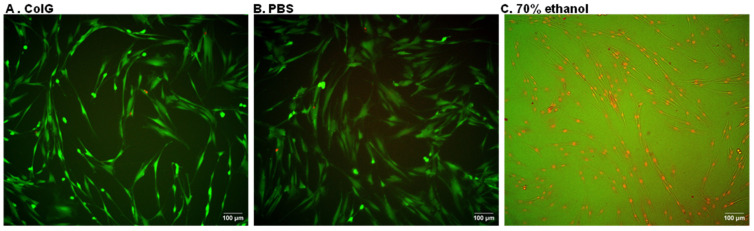
**Cellular toxicity.** hGFs treated with (**A**) 5 mg/mL ColG, (**B**) PBS or (**C**) 70% ethanol. Green cells are viable and red cells are dead. The indicated scale bar shows 100 μm length.

As a final validation that the enzyme is indeed not toxic to the cells, we monitored the cellular death of hGFs. To this end, cells were seeded in a 6-well plate, 1,200,000 cells/well, and treated with ColG at a concentration of 5 mg/mL, PBS and 70% ethanol. Following overnight incubation, cellular images were recorded. Figure 6 shows images of cells with the three treatments. As it can be observed, collagenase does not impart cellular death on the hGFs.

## 4. Discussion

The main objective of the present study was to explore for the first time the effect of an enzymatic disruption of the PDL fibers on the force required for tooth extraction. The integration of the novel extraction model with the innovative biological treatment described in this paper enabled the analytical determination of the force required for the extraction of the roots of the first and second split porcine mandibular premolars, with and without enzymatic treatment. Indeed, applying ColG had an immense effect of up to 50% reduction in the force required for the root extraction.

Reducing the forces required for tooth extraction is a long-term challenge in dental medicine and has important implications for reducing intra- and post-operative complications, particularly in medically compromised patients, such as those with hemostasis disorders or those treated with antiresorptive (e.g., bisphosphonates) or antiangiogenic agents [34,35]. Atraumatic or minimally invasive extraction is also important for successful prosthetic rehabilitation with dental implants, since it facilitates the preservation of adjacent hard and soft tissues. Such a procedure also provides a general approach towards non-invasive medicine by reducing the need for flap surgery. Moreover, significant and reliable technological advancements in the field of exodontia may positively affect the operator’s confidence and thus facilitate the general dental practitioners’ ability to carry out complicated tooth extractions. Such progress may diminish patient referrals to secondary care settings, such as an oral surgery unit, and also decrease patients’ stress and anxiety levels. Finally, healthy extracted teeth may be preserved and serve as autogenous tooth bone graft material for many situations that demand osseous grafts [36].

Furthermore, the present study established a generalized and robust model for recording the force applied during the tooth extraction process. The latter comprises an important contribution to the scholarship aimed at understanding PDL structure and function [28,37,38,39,40,41,42,43]. Our setup is generic and can be extended to a broad range of research applications.

Although rarely studied, the average force required to extract a human tooth from the alveolar socket ranges from 40 to 600 newton (N), with significant variability between different types of roots [32,37,40]. 

In our model, the mean force required to extract a split root of porcine mandibular PM1 or PM2 was in the range of around 200 N, which is roughly comparable to that of human teeth.

Given the high efficiency of ColG and its capability to fully degrade collagen fibers into short peptide fragments, our expectation was to observe a large and relatively uniform impact across different jaws. However, great variability was measured. Interestingly, in cases in which only minor differences were observed between ColG and PBS treated roots, the counterpart teeth were mostly from the same jaw. This finding may suggest that the lack of difference in the applied force after ColG treatment may result from biological reasons that are attributed to structural factors and viscoelasticity of the PDL in the specific jaw [37,38,40,44]. However, explaining the observed behavior solely by the mechanical response of the PDL is an over-simplification. Indeed, additional tooth-related factors, such as curvature and divergence of the roots, may also impact the force required for the extraction.

Although harnessing natural or engineered biological machinery, such as enzymes, is an attractive approach to treat a broad range of indications in general medicine [45,46], the literature includes only a limited number of studies concerning the oral cavity, particularly in regard to the enzymatic degradation of the PDL [22,47,48]. However, the implementation of collagenase in oral and maxillofacial medicine may be broadly applicable beyond minimally invasive tooth extraction, since the enzymatic degradation of collagen may potentially have clinical relevance for the treatment of post-surgical scars and hypertrophic lesions in the oral mucosa, such as irritation fibroma and gingival fibromatosis; prevention of post-orthodontic treatment relapse (by replacing the use of a surgical scalpel for the procedure known as circumferential supracrestal fiberotomy); non-surgical crown lengthening procedures; and even the decontamination of the tooth or implant surface.

We also note the limitations of the current ex vivo study, such as safety and patient chair-time, in regard to the clinical setting. Concerning safety, even though the application of clostridial collagenase G (Xiaflex®) is approved by the United States Food and Drug Administration (FDA) for the treatment of Dupuytren’s and Peyronie’s diseases, it is associated with several adverse effects, including bruising/hematomas [48]. In addition, at concentrations ranging from 250 to 1000 ng/mL, Xiaflex® has been shown to negatively affect fat and skin fibroblast proliferation, due to the induction of membrane leakage [33]. However, following drug retrieval, cells exhibited recovery. This finding is congruent with our current cellular viability assays testing the effect of ColG on hGFs. Ultimately, comprehensive efforts will need to be undertaken to evaluate the adverse effects of this enzyme within the oral cavity on the surrounding tissues and adjacent roots. Furthermore, the effects of collagenase on the eventual alveolar bleeding should be evaluated before clinical studies in humans. Yet, considering that clostridial collagenase G was found to be safe in vivo, the implementation of the recombinant ColG in the oral cavity is a promising approach for further testing.

Another issue is the time span required from the injection of the enzyme until a tooth extraction step. In the current setup, an incubation period of sixteen hours was empirically selected. In practice, such a procedure would demand two consecutive visits, fairly doubling the patient chair-time. In addition, as opposed to the ex vivo model, living tissues possess immune and metabolic activities that may further dilute the effect of collagenase. Therefore, optimized protocols should be established, aiming to adjust the volume, concentration and buffer formulation for the optimal stability and activity of the enzyme, preferably shortening the treatment duration.

Furthermore, these protocols should be further validated in vivo to pave the way for the application of the enzymatically assisted tooth extraction in the clinical practice. Likewise, other bacterial collagenases should be tested, which are known to affect the desired response differently [23,27,49,50,51]. Furthermore, direct enzyme evolution can entail screening for an optimized enzyme by imparting specific point mutations that will improve enzyme solubility, stability, and most importantly activity [29].

## 5. Conclusions

In this paper, we provided the first evidence for enzymatically assisted tooth extraction. Even though there is still a long way for it to be part of an accepted clinical protocol, this novel technique brings us closer to minimally invasive tooth extraction, potentially reducing intra- and post-operative complications. There are still various factors to consider for further advancing it from bench to bedside. An optimal protocol for the time and dose of injection before extraction, exploration of tooth type that will benefit from such an application, and the careful clinical evaluation of post-extraction outcomes must be executed. These, of course, must first be evaluated in vivo in a suitable animal model. Additionally, factors related to the operator, such as storage conditions and cost, must be thought through. Following in vivo validation, clinical trials showing the safety and benefit will need to be further executed. It is important to note that, beside tooth extraction, the enzymatic degradation of collagen can advance a broad range of diverse applications in dental medicine. These include immediate implant placement, the treatment of post-surgical scars and hypertrophic lesions in the oral mucosa, such as irritation fibroma and gingival fibromatosis; the prevention of post-orthodontic treatment relapse (by replacing the use of a surgical scalpel, known as circumferential supracrestal fiberotomy); non-surgical crown lengthening procedures; and even the decontamination of the tooth or implant surface. Lastly, a synergistic application could be developed in which currently existing minimally invasive extraction techniques would complement each other. Such a development could lead to the optimized reduction of the force required for tooth extraction and enhance progress towards the long-sought-after atraumatic operations in dental medicine.

## Data Availability

The datasets analyzed during the current study are available from the corresponding authors upon reasonable request.
